# Image-Based Differentiation of Intracranial Metastasis From Glioblastoma Using Automated Machine Learning

**DOI:** 10.3389/fnins.2022.855990

**Published:** 2022-05-12

**Authors:** Yukun Liu, Tianshi Li, Ziwen Fan, Yiming Li, Zhiyan Sun, Shaowu Li, Yuchao Liang, Chunyao Zhou, Qiang Zhu, Hong Zhang, Xing Liu, Lei Wang, Yinyan Wang

**Affiliations:** ^1^Department of Neurosurgery, Beijing Tiantan Hospital, Capital Medical University, Beijing, China; ^2^Beijing Neurosurgical Institute, Capital Medical University, Beijing, China; ^3^China National Clinical Research Center for Neurological Diseases, Beijing, China; ^4^Chinese Institute for Brain Research, Beijing, China

**Keywords:** automated machine learning, glioblastoma, intracranial metastasis, image-based differentiation, prediction

## Abstract

**Purpose:**

The majority of solitary brain metastases appear similar to glioblastomas (GBMs) on magnetic resonance imaging (MRI). This study aimed to develop and validate an MRI-based model to differentiate intracranial metastases from GBMs using automated machine learning.

**Materials and Methods:**

Radiomics features from 354 patients with brain metastases and 354 with GBMs were used to build prediction algorithms based on T2-weighted images, contrast-enhanced (CE) T1-weighted images, or both. The data of these subjects were subjected to a nested 10-fold split in the training and testing groups to build the best algorithms using the tree-based pipeline optimization tool (TPOT). The algorithms were independently validated using data from 124 institutional patients with solitary brain metastases and 103 patients with GBMs from the cancer genome atlas.

**Results:**

Three groups of models were developed. The average areas under the receiver operating characteristic curve (AUCs) were 0.856 for CE T1-weighted images, 0.976 for T2-weighted images, and 0.988 for a combination in the testing groups, and the AUCs of the groups of models in the independent validation were 0.687, 0.831, and 0.867, respectively. A total of 149 radiomics features were considered as the most valuable features for the differential diagnosis of GBMs and metastases.

**Conclusion:**

The models established by TPOT can distinguish glioblastoma from solitary brain metastases well, and its non-invasiveness, convenience, and robustness make it potentially useful for clinical applications.

## Introduction

Metastatic tumors (Schiff, 2001) may be difficult to distinguish from glioblastomas (GBMs; Jiang et al., 2016). Neurological symptoms are the initial clinical manifestation in 10–30% of patients with brain metastases and are also observed in many patients with GBM (Schiff, 2001). Moreover, solitary brain metastases appear similar to GBMs on magnetic resonance imaging (MRI). Differentiating the two types of cancer is important because they require different treatment approaches (National Guideline, 2018), and early detection of metastases can facilitate the detection of primary lesions in asymptomatic patients.

Several imaging techniques have been used to distinguish brain metastases from GBMs, including magnetic resonance spectroscopy (Ishimaru et al., 2001; Opstad et al., 2004; Tsougos et al., 2012), dynamic susceptibility contrast-enhanced (CE) scanning (Cha et al., 2007; Blasel et al., 2010; Ma et al., 2010; Server et al., 2011; Lehmann et al., 2012; Tsougos et al., 2012; Bauer et al., 2015; Askaner et al., 2019; She et al., 2019), diffusion tensor imaging (Byrnes et al., 2011), diffusion-weighted imaging (Byrnes et al., 2011), and three-dimensional-arterial spin labeling (Lin et al., 2016). With the development of radiomics and extraction technology, texture features are increasingly used to distinguish between GBM and metastases. For example, one algorithm based on *k*-means clustering of nine texture features extracted from structural images and data on cerebral blood volume has been shown to differentiate the two types of cancer with 92% sensitivity and 71% specificity (Mouthuy et al., 2012). In a recent study, an algorithm based on gray-scale texture features outperformed the algorithm based on shape features (Petrujkić et al., 2019).

Machine learning can use high-throughput radiomics information to identify different neoplastic diseases (Petrujkić et al., 2019). In one study, information extracted from dynamic magnetic sensitivity CE scanning was able to differentiate GBMs from metastases with 98% accuracy (Bauer et al., 2015). In another study (Chen et al., 2013), Bayesian network-based decision support systems were used to differentiate GBMs from solitary metastases with 94% accuracy and an area under the receiver operating characteristic (ROC) curve (AUC) of 0.90. A third study (Tsolaki et al., 2013) found that a support vector machine (SVM) approach achieved higher accuracy (98%) than approaches based on naive Bayes or *k*-nearest neighbor. A neural network-based classifier (Yang et al., 2014) achieved 98% accuracy and 0.975 AUC. Although these results are promising, the clinical applicability of these algorithms is limited by the relatively small sample size and lack of validation.

The use of machine learning in previous studies significantly requires manual work to determine the optimal combination of feature selection methods and classifiers. Automatic machine learning can automate these processes and theoretically achieve optimal model performance based on the training data. The current study developed a set of algorithms based on high-throughput radiomics information from MRI to differentiate brain metastases from GBMs. Feature selection, classifier selection, and parameter optimization were conducted using a tree-based pipeline optimization tool (TPOT; Le et al., 2019) that relies on genetic algorithms to optimize machine learning pipelines. The models built by the pipelines were validated both internally and externally.

## Materials and Methods

### Patient Cohorts

**FIGURE 1:**
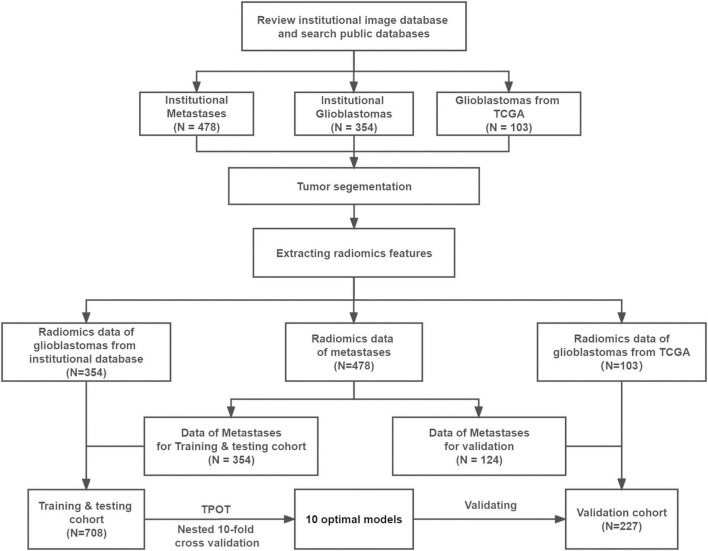
Study flowchart. TCGA, the cancer genome atlas; TPOT, tree-based pipeline optimization tool.

The cohort used for training and testing included 354 patients with brain metastases who received treatment between 2008 and 2016 at Tiantan Hospital (Beijing, China) and 354 patients with GBMs who received treatment between 2015 and 2017 at the same hospital (Figure 1). In all patients, the diagnosis was confirmed by histopathology of the surgically resected specimens. To be included in the study, patients had to be at least 18 years old at diagnosis, and preoperative T2-weighted and CE T1-weighted MRI data had to be available. Patients with lesions involving the saddle area, skull, and/or scalp were excluded.

The validation cohort comprised 124 time-independent patients with brain metastases who received treatment from 2016 to 2018 at Tiantan Hospital and 103 external patients with GBMs from the cancer genome atlas (TCGA).

This study was approved by the Ethics Committee of Tiantan Hospital. All data were retrospectively collected from an institutional medical database or extracted from public databases, and the need for informed consent was waived by the ethics committee.

### Imaging Data Acquisition

Magnetic resonance imaging was performed using a Magnetom Trio 3T MR scanner (Siemens Healthcare, Erlangen, Germany) in all patients with brain metastases. For the 354 patients with GBM at the Tiantan Hospital, Magnetom Trio 3T MR scanner was used in the 253 patients. In the remaining 101 patients, MRI was performed using Sigma 3T MR scanner (General Electric Company). For T2-weighted images, the specifications were as follows: repetition time, 4,500–6,000 ms; echo time, 84–122.5 ms; slice thickness, 3–5 mm; field of view, (180–240) mm × (219–256) mm; and matrix size, (160–512) × (208--512) pixels. For CE images, enhancement was achieved by injecting gadolinium-diethylenetriamine penta-acetic acid (0.1 mmol/kg, Beijing Beilu Pharmaceutical, Beijing, China), and the acquisition parameters were as follows: repetition time, 560--2520 ms; echo time, 2.3--19.7 ms; and slice thickness, 3--5 mm. The MR images of GBMs from the TCGA database were downloaded from the Cancer Imaging Archive.^1^

Lesions were manually segmented on T2-weighted images (tumor and peritumoral edema area) and CE images (tumor area) by two neurosurgeons using MRIcro.^2^ Segmentation was evaluated by a neuroradiologist with more than 20 years of experience in brain tumor diagnosis.

### Extraction of Radiomics Information

Before radiomics feature extraction, an intensity standardization (*z*-score transformation) was performed to minimize intensity drift across different MRI images, and MRI voxels were resampled to 1 mm × 1 mm × 1 mm to overcome heterogeneous slice thickness and resolution. PyRadiomics (Griethuysen et al., 2017) was used to acquire radiomics features, and 1,510 features (Supplementary Table 1) were extracted. To reduce the bias caused by different data sources, Student’s *t*-test was used to identify the features that were significantly different between the institutional GBM patients and GBM patients of TCGA, and these features were excluded. Finally, 492 features extracted from CE T1-weighted images and 440 features extracted from T2-weighted images were included and used separately and jointly for model building.

### Automated Machine Learning and Statistical Analysis

Tree-based pipeline optimization tool^3^ is based on the open-source software library Scikit-learn (Pedregosa et al., 2012) on Python, and its default configuration, which included 13 classifiers, and 7 feature selection or decomposition methods (Supplementary Table 2), was used in this study. TPOT iteratively using a genetic algorithm to optimized pipeline and was conducted for 100 generations with a population size of 100. The score of each pipeline being optimized was evaluated in cross-validation and finally the pipeline with highest score was exported. In order to make the results more interpretable, the form of the pipeline was set to “Selector-Transformer-Classifier” which means each pipeline includes feature selection, data transformation, and fitting of classifier in turn.

**FIGURE 2:**
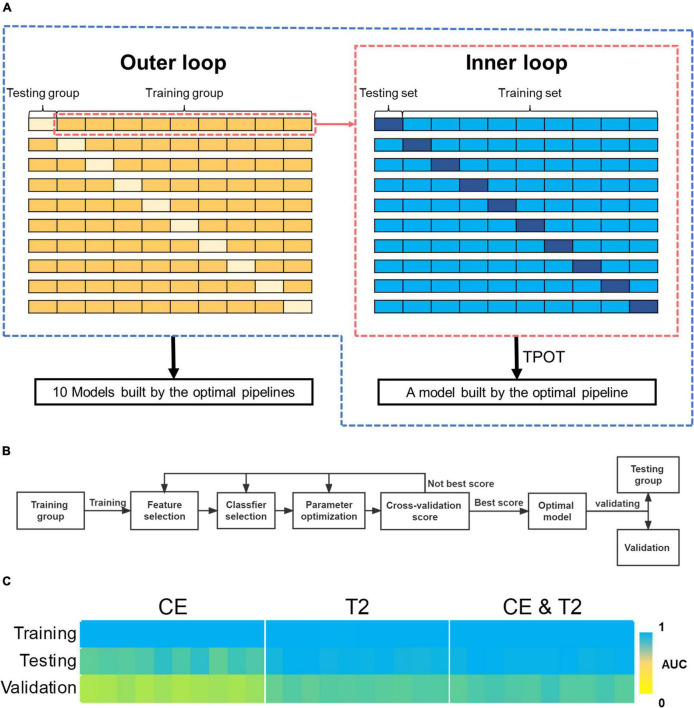
The process of automating machine learning and the predictive performance of the models it builds. **(A)** The two-layer loops for 10-fold nested cross-validation. **(B)** The process of automating machine learning modeling and validation using TPOT. **(C)** AUCs for the models built by the 10-fold nested cross-validation during training, testing, and validation. AUC, areas under the receiver operating characteristic curve; TPOT, tree-based pipeline optimization tool.

The cohort used for training and testing was shuffled first. Subsequently, a nested 10-fold cross-validation that contains two layers of loops were used in the analysis: A 10-fold split was used to divide all the samples into 10 groups of samples. Each group was defined as a testing group in turn, and the remaining groups were defined as a training group; therefore, 10 training and testing groups were constructed. TPOT was used to drive automated machine learning for each training group and to optimize the exported pipeline. In this process, a 10-fold cross-validation was used to optimized the configuration of pipeline based on diagnostic accuracy. This process was defined as “inner loop,” and a model built by the optimal pipeline would be exported after an inner loop was completed. Each testing group was used as independent data to test the performance of the model that was trained using the corresponding training group. The process of performing TPOT process in turn for each training group was defined as “outer loop.” Finally, 10 models built by the optimal pipelines would be exported after the nested 10-fold cross-validation process was completed (Figure 2). These 10 models were utilized as a group of models to predict the validation cohort, and the probability of predictions was weighted by the probability values predicted by each model in the group.

The performance of models and groups of models was assessed in terms of accuracy, precision, sensitivity, specificity, and AUC. The chi-squared test was used to compare categorical variables, and Student’s *t*-test was used to compare continuous variables in different subgroups.

### Assessment by Neuroradiologists

The T2-weighted and CE images of the patients in the validation cohort were independently reviewed by two neuroradiologists with at least 5-year experience using a 5-point scale (one for definitive metastasis and five for definitive GBM). The assessors were blinded to clinical and pathological data. The performance of human readers was evaluated by ROC analysis.

## Results

### Patient Demographics

The cohort for training and testing comprised 354 patients with metastases (197 men and 157 women, mean age 53.6 ± 10.4 years) and 354 patients with GBMs (206 men and 148 women, mean age 49.9 ± 15.6 years), respectively. The validation cohort comprised 124 patients with metastases (64 men and 60 women, mean age 56.6 ± 8.8 years) and 103 patients with GBMs (56 men and 47 women, mean age 59.2 ± 14.3 years). The two cohorts did not differ significantly in sex (*p* = 0.25) and cancer types (*p* = 0.23). However, patients with metastasis were significantly older (54.3 ± 10.1 vs. 52.1 ± 15.8 years, *p* = 0.01). Patients in the validation cohort were older than those in the training and testing cohorts (56.3 ± 12.7 vs. 52.3 ± 13.2 years, *p* < 0.01; Table 1).

**TABLE 1:** Age and sex distribution.

	Overall *N* = 935	Metastases *N* = 478	Glioblastomas *N* = 457	*p*	Training and testing *N* = 708	Validation *N* = 227	*p*
Age (year ± SD)	53.2 ± 13.2	54.3 ± 10.1	52.1 ± 15.8	0.01	52.3 ± 13.2	56.3 ± 12.7	<0.01
Sex (*n*, ratio)				0.34			0.25
Male	523 (0.56)	261 (0.55)	262 (0.57)		403 (0.57)	120 (0.53)	
Female	412 (0.44)	217 (0.45)	195 (0.43)		305 (0.43)	107 (0.47)	

*SD, standard deviation.*

### Optimal Machine Learning Pipelines

A total of 30 pipelines were generated: 10 for each type of data input (T2-weighted, CE, or both). The AUCs obtained for various pipelines are shown in Table 2.

**TABLE 2:** Areas under the receiver operating characteristic curves for the 30 predictive models during training, testing, and validation.

Radiomics features	Cohort	Models trained by different training groups										Average
		1	2	3	4	5	6	7	8	9	10	

CE	Training	1.000	1.000	1.000	1.000	1.000	1.000	1.000	1.000	1.000	1.000	1.000
	
	Testing	0.817	0.841	0.835	0.848	0.911	0.854	0.914	0.816	0.879	0.848	0.856
	
	Validation	0.661	0.671	0.700	0.665	0.692	0.663	0.679	0.691	0.673	0.698	0.679

T2	Training	1.000	1.000	1.000	0.999	1.000	1.000	1.000	1.000	1.000	1.000	1.000
	
	Testing	0.959	0.998	0.994	0.969	0.982	0.977	0.988	0.964	0.958	0.973	0.976
	
	Validation	0.802	0.839	0.812	0.837	0.830	0.838	0.824	0.822	0.840	0.837	0.828

CE and T2	Training	1.000	1.000	1.000	1.000	1.000	1.000	1.000	1.000	1.000	1.000	1.000
	
	Testing	0.973	1.000	0.998	0.986	0.986	0.986	0.999	0.975	0.984	0.990	0.988
	
	Validation	0.817	0.848	0.864	0.847	0.838	0.882	0.834	0.835	0.864	0.826	0.846

*CE, contrast-enhanced T1-weighted.*

**FIGURE 3:**
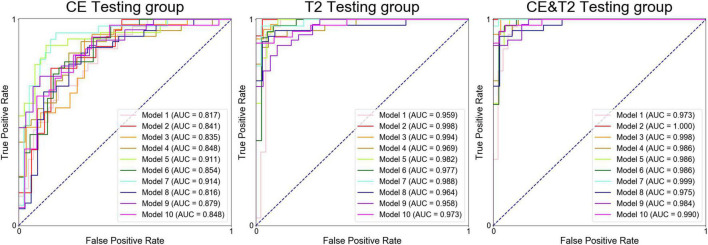
Receiver operating characteristic curves for the models during the corresponding testing groups.

All prediction models performed reasonably well during training. The AUCs of 29 models reached 1.000 in the training group, whereas the AUC of model 4 based on T2-weighted features was 0.999. The ROC curves for the corresponding testing groups are shown in Figure 3.

Models based on CE features performed reasonably well during testing but poorly during validation. The average AUCs were 0.856 (95% confidence interval: 0.832–0.881) and 0.679 (0.669–0.690) in the testing and validation cohorts, respectively. The models based on T2-weighted features performed well during testing and validation. The average AUCs were 0.976 (0.966–0.986) and 0.828 (0.819–0.837) in the testing and validation cohorts, respectively. The models based on the combination of CE and T2-weighted features had average AUCs of 0.988 (0.981–0.994) and 0.846 (0.831–0.860) in the testing and validation cohorts, respectively.

The performance of the models based on CE features was significantly lower than that based on the T2-weighted features in the testing group (average AUC 0.856 vs. 0.976, *p* < 0.01) and the validation group (average AUC 0.679 vs. 0.828, *p* < 0.01). The models based on the combination of CE and T2-weighted features had a higher average AUC than those based on CE or T2-weighted features in the testing and validation groups.

**FIGURE 4:**
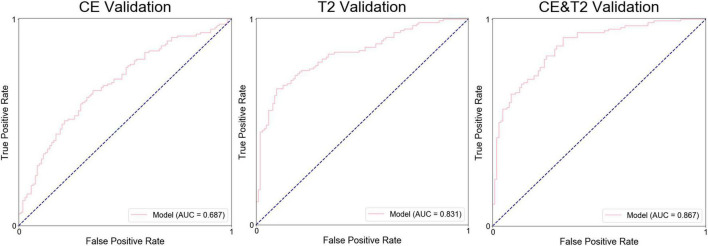
Receiver operating characteristic curves for the groups of models during validation.

The average prediction probability obtained from the prediction in the validation by a group of models was used for the ROC analysis to determine the performance of the group of models. The AUC of the groups of models based on CE features, T2-weighted features, and the combination were 0.687, 0.831, and 0.867, respectively, (Figure 4). The accuracy, precision, specificity, and sensitivity of the groups of models are shown in Table 3.

**TABLE 3:** The predicting performance of the groups of models in the validation cohort.

Models based on	Accuracy	Precision	Specificity	Sensitivity	AUC
CE features	0.656	0.698	0.660	0.653	0.687
T2 features	0.749	0.768	0.718	0.774	0.831
CE and T2 features	0.784	0.805	0.767	0.798	0.867

*CE, contrast-enhanced T1-weighted.*

### Key Features Selected in the Best Models

**FIGURE 5:**
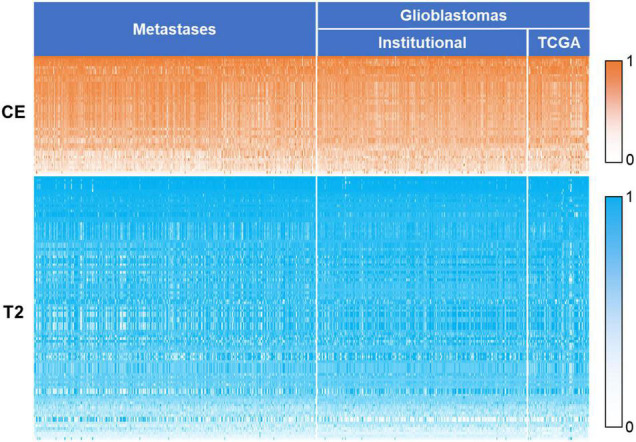
Heatmap of the 149 key features. The values of the features were normalized using min–max normalization. The values of contrast-enhanced T1-weighted features are represented by white to orange (0–1), and values of T2-weighted features are represented by white to blue. CE, contrast-enhanced T1-weighted; TCGA, the cancer genome atlas.

To identify generalizable features allowing accurate diagnostic prediction, we reviewed the selected features from all 10 models based on a combination of CE T1-weighted and T2-weighted features, which had the highest average AUC. The pipelines for these 10 models are listed in Supplementary Table 3. There were 149 features that were included in all 10 models, of which 46 were CE and 103 were T2-weighted features (Table 4): 3 shape features, 48 first-order original or derived features, 34 GLCM original or derived features, 27 GLRLM original or derived features, 20 GLSZM-derived features, and 17 GLDM-derived features. The details of these features are shown in Supplementary Table 4, and their heatmaps are shown in Figure 5.

**TABLE 4:** The distribution of features included in all model of the best algorithms.

Features	CE		T2-weighted		Total
Shape	2		1		3
	Original	Derived^a^	Original	Derived	0
First-order	3	23	0	22	48
Gray-level co-occurrence matrix (GLCM)	0	7	1	26	34
Gray-level run-length matrix (GLRLM)	0	7	3	17	27
Gray-level size zone matrix (GLSZM)	0	2	0	18	20
Gray-level dependence matrix (GLDM)	0	2	0	15	17
Total	46		103		149

*CE, contrast-enhanced T1-weighted.*

*^a^Derived features include exponential, gradient, logarithm, log sigma, square, square root, and wavelet features derived from the original features.*

### Comparison Between Human Reader and Prediction Model

In the validation cohort, the AUC, accuracy, precision, specificity, and sensitivity obtained by neuroradiologist 1 were 0.628, 0.658, 0.597, 0.789, and 0.547, respectively. For neuroradiologist 2, the AUC, accuracy, precision, specificity, and sensitivity were 0.513, 0.586, 0.537, 0.734, and 0.461, respectively. The best group of models clearly outperformed the human readers (AUC 0.867 vs. 0.628 vs. 0.513 in the validation cohort).

## Discussion

In our study, radiomics features were extracted from CE and T2-weighted MRI. Features extracted from the two sequences of images were applied alone or in combination to generate predictive models using automated machine learning. Several models performed well in distinguishing metastases from GBMs during testing and validation. The group of models based on a combination of contrast T1-weighted and T2-weighted features performed best, and 149 features were identified as the most important for the differentiation of brain metastases from GBMs.

Various models based on radiomics and machine learning were used to differentiate metastases from GBMs in previous studies, but had limitations of relatively small sample sizes and lack of external validation. For example, an SVM classifier (Artzi et al., 2019) based on CE features showed an accuracy of 0.8, whereas the “least absolute shrinkage and selection operator” for feature selection and SVM classifier for model establishment (Qian et al., 2019) showed an AUC of 0.90 during testing, but no validation was reported. In the current study, a cohort of data from an institution was used for training and testing, whereas external data from TCGA were combined with time-independent institutional data for validation.

The present study used TPOT to automate feature selection, classifier selection, and parameter optimization. TPOT was previously applied to predict H3 K27M mutations in brain midline gliomas (Su et al., 2019), achieving an AUC of 0.903 during testing. TPOT also showed excellent ability to generate models that accurately diagnose coronary heart disease based on angiographic data (Orlenko et al., 2019) or to predict the age of the brain based on brain images (Dafflon et al., 2020). In the current study, the differentiation of metastases from GBMs using TPOT achieved a higher accuracy in testing (average AUC = 0.988) than in previous studies using other classifiers (Tsolaki et al., 2013; Artzi et al., 2019; Petrujkić et al., 2019; Qian et al., 2019). Similar to previous studies (Qian et al., 2019; Bae et al., 2020), machine learning models based on radiomics outperformed human readers.

Deep learning-based technologies were also applied to distinguish glioblastoma from brain metastases. In a previous study, a model combining convolutional neural network-based features and radiomics features was found to be more effective in distinguishing these two tumors than the model using only radiomics features (The AUC in the test set improved from 0.93 to 0.97; Liu et al., 2021). In another study, deep learning was used to differentiate glioblastoma and solitary brain metastases with AUCs of 0.889 and 0.835 in the training and test sets, respectively (Shin et al., 2021). The prediction performance of these deep learning-based models is not superior to our model.

In the present study, radiomics features extracted from T2-weighted images were more effective for tumor differentiation than features extracted from CE images. This result is consistent with that in a previous study (Petrujkić et al., 2019), which only included 14 morphometric parameters extracted from images, and we further confirmed this result with more features. The explanation might be that T2-weighted images provide more information about features related to heterogeneous angiogenesis (Hopewell et al., 1993). T2-weighted images can also reveal information about edema; thus, it may differentiate the purely vasogenic edema associated with brain metastases from a mixture of vasogenic edema and tumor cell infiltration associated with GBMs (Artzi et al., 2018).

We identified 149 features as potentially the most effective for distinguishing GBMs and brain metastases. Shape features included descriptors of the size and shape of the segmented lesions. Sphericity based on CE images is a feature describing the spherical nature of the tumor, and in the current study, GBMs were more spherical than metastases (*p* < 0.01). Elongation based on CE images showed that the tumor was elongated, and metastases were more elongated than GBMs (*p* < 0.01). Flatness based on T2-weighted images shows the flatness of the area of tumor and peritumoral edema, and lesions of GBMs were flatter than those of metastases (*p* < 0.01). First-order features describe the distribution of voxel intensities. For example, maximum and its derived features show the maximum gray-level intensity within the lesion area, and the result showed that the maximum gray-level intensity of metastases was significantly higher than that of GBMs on CE images (*p* < 0.01) but lower on T2-weighted images (*p* < 0.01). The other features are texture features or features derived from texture features and are calculated from GLCM, GLSZM, GLRLM, and GLDM, respectively. They described the patterns or spatial distributions of voxel intensities. Most of the included texture features were based on the T2 images (76/94). Texture features extracted from T2-weighted images provide information about ischemia, edema, and necrosis inside the tumor region (Chen et al., 2015). Our analysis highlights the power of texture features to differentiate GBMs and brain metastases, in part because they can capture the greater tumor-area heterogeneity of metastases on CE T1-weighted images and the greater tumoral and peritumoral area heterogeneity of GBMs on the T2-weighted images.

Our study has some limitations. One is the lack of external validation data for brain metastases, since we were unable to find publicly available data. In the future, multicenter imaging data on GBMs and metastases should be used to verify the robustness of the TPOT-generated models. Additionally, the radiomics information included in this study was only extracted from CE T1-weighted and T2-weighted images, and multimodal image data may be added to the model to further improve the efficiency of classification in the future.

An automated machine learning algorithm was used to fit the models for image-based differentiation of intracranial metastases from GBMs. Models based on CE features proved unsuitable for this task, whereas models based on T2-weighted features performed significantly better. The optimal group of models, which was based on a combination of CE and T2-weighted features, had an AUC of 0.867 during validation. This group of models may be able to help different GBMs and intracranial metastasis in a timely and non-invasive manner before surgery and then develop a more appropriate treatment plan.

## Data Availability Statement

The original contributions presented in the study are included in the article/Supplementary Material, further inquiries can be directed to the corresponding authors.

## Ethics Statement

The studies involving human participants were reviewed and approved by the Ethics Committee of Tiantan Hospital. Written informed consent for participation was not required for this study in accordance with the national legislation and the institutional requirements.

## Author Contributions

YKL participated in the writing of the manuscript, data collection, tumor segmentation, data processing and analysis, and completed the rest of the manuscript and the final draft. TL mainly participated in the writing of abstract, methods, and discussion parts and the revision of the second draft of this manuscript. ZF, YL, ZS, SL, YCL, and CZ gave important technical support in data process and bug detecting. QZ, HZ, and XL gave us many help in polishing and modifying of this article. All authors contributed to the article and approved the submitted version.

## Conflict of Interest

The authors declare that the research was conducted in the absence of any commercial or financial relationships that could be construed as a potential conflict of interest.

## Publisher’s Note

All claims expressed in this article are solely those of the authors and do not necessarily represent those of their affiliated organizations, or those of the publisher, the editors and the reviewers. Any product that may be evaluated in this article, or claim that may be made by its manufacturer, is not guaranteed or endorsed by the publisher.

## Footnotes

1
http://www.cancerimagingarchive.net/

2
http://www.mccauslandcenter.sc.edu/mricro/

3
http://epistasislab.github.io/tpot/

## Abbreviations

MRI: magnetic resonance imaging
TPOT: Tree-based pipeline optimization tool
GBMs: glioblastomas
ROC: receiver operating characteristic
SVM: support vector machine
CE: contrast-enhanced
TCGA: The Cancer Genome Atlas.
