# Supplementary material for: Image-Based Differentiation of Intracranial Metastasis From Glioblastoma Using Automated Machine Learning
Source: Front Neurosci. 2022 May 12;16:855990. doi: 10.3389/fnins.2022.855990 (PMC9133479; doi:10.3389/fnins.2022.855990)
Supplement: Supplementary file 1 [file Data_Sheet_1.pdf]

Supplemental Table 1. The detail of 1510 features extracted by PyRadiomics.

Features	Total
**Shape features**	14
**First-order features**	18
**Gray-level co-occurrence matrix features**	24
**Gray-level run-length matrix features**	·16
**Gray-level size zone matrix features**	16
**Gray-level dependence matrix features**	14
**Log sigma features**	264
**Wavelet features**	704
**square features**	88
**square root features**	88
**logarithm features**	88
**exponential features**	88
**gradient features**	88
**Total**	1510

Information of these features and the formulas for calculating them can be found in the documentation of PyRadiomics (https://pyradiomics.readthedocs.io/en/latest/features.html).

Supplemental Table 2. Classifier and feature selector or decomposition method included in TPOT.

**Classier**	**Feature selector or decomposition method**
Gaussian naive Bayes	Select the p-values corresponding to the family-wise error rate (SelectFwe)
Bernoulli naive Bayes	Select features according to a percentile of the highest scores (SelectPercentile)
Multinomial naive Bayes	Feature selector that removes all low-variance features (VarianceThreshold)
Decision tree	Feature ranking with recursive feature elimination (RFE)
Extra trees	Meta-transformer for selecting features based on importance weights (SelectFromModel)
Random forest	Fast algorithm for independent component analysis (FastICA)
Gradient boosting	Principal component analysis (PCA)
K-nearest neighbors	
Linear support vector classification	
Logistic regression	
Extreme gradient boosting	
Stochastic gradient descent	
Multi-layer perceptron	

TPOT (version 0.23.2) based on scikit-learn (version 0.23.2) were used in this study.

Supplemental Table 3. Pipelines in the tree-based pipeline optimization tool using combination of contrast-enhanced T1 weighted and T2 weighted features.

Pipeline	Feature selector	Number of selected features	Transformer	Classfier
**1**	VarianceThreshold	624	MinMaxScaler	GradientBoosting
**2**	RFE	466	OneHotEncoder	
**3**	RFE	466	OneHotEncoder	
**4**	RFE	466	MaxAbsScaler	
**5**	RFE	466	MinMaxScaler	
**6**	RFE	466	RobustScaler	
**7**	RFE	466	OneHotEncoder	
**8**	VarianceThreshold	622	RobustScaler	
**9**	RFE	466	StandardScaler	
**10**	RFE	466	MinMaxScaler	

Abbreviation: RFE, Feature ranking with recursive feature elimination.

Supplemental Table 4. Details of the 149 key features.

Contrast-enhanced T1-weighted features	T2-weighted features
**Original features**	
original_firstorder_Kurtosis	original_glcm_Imc2
original_firstorder_Median	original_gldm_DependenceVariance
original_firstorder_Skewness	original_glrlm_GrayLevelNonUniformity
original_shape_Elongation	original_glrlm_LongRunLowGrayLevelEmphasis
original_shape_Sphericity	original_glrlm_RunLengthNonUniformity
	original_shape_Flatness
**Exponential features**	
exponential_firstorder_RootMeanSquared	exponential_firstorder_Kurtosis
	exponential_glrlm_GrayLevelNonUniformity
**Gradient features**	
gradient_firstorder_InterquartileRange	gradient_glrlm_RunLengthNonUniformity
gradient_glszm_GrayLevelNonUniformity	
**Logarithm features**	
logarithm_firstorder_Maximum	logarithm_firstorder_Entropy
logarithm_firstorder_Mean	logarithm_firstorder_Range
logarithm_firstorder_Median	logarithm_firstorder_Uniformity
logarithm_firstorder_RootMeanSquared	logarithm_glcm_Autocorrelation
logarithm_glrlm_ShortRunHighGrayLevelEmphasis	logarithm_glcm_ClusterTendency
logarithm_glszm_LowGrayLevelZoneEmphasis	logarithm_glcm_DifferenceEntropy
	logarithm_glcm_Imc2
	logarithm_glcm_JointAverage
	logarithm_glcm_JointEnergy
	logarithm_glcm_JointEntropy
	logarithm_glcm_SumAverage
	logarithm_glcm_SumEntropy
	logarithm_glcm_SumSquares
	logarithm_gldm_GrayLevelVariance
	logarithm_gldm_HighGrayLevelEmphasis
	logarithm_glrlm_GrayLevelNonUniformity
	logarithm_glrlm_LongRunLowGrayLevelEmphasis
	logarithm_glrlm_ShortRunEmphasis
	logarithm_glszm_HighGrayLevelZoneEmphasis
**Log-sigma features**	
log-sigma-3-0-mm-3D_glrlm_LongRunLowGrayLevelEmphasis	log-sigma-3-0-mm-3D_firstorder_90Percentile
log-sigma-3-0-mm-3D_glrlm_RunVariance	log-sigma-3-0-mm-3D_firstorder_Kurtosis
log-sigma-4-0-mm-3D_firstorder_Skewness	log-sigma-3-0-mm-3D_firstorder_Maximum
log-sigma-4-0-mm-3D_glrlm_ShortRunEmphasis	log-sigma-3-0-mm-3D_firstorder_Skewness
log-sigma-5-0-mm-3D_glrlm_ShortRunEmphasis	log-sigma-3-0-mm-3D_glrlm_HighGrayLevelRunEmphasis
	log-sigma-4-0-mm-3D_firstorder_Maximum
	log-sigma-4-0-mm-3D_gldm_GrayLevelNonUniformity

	log-sigma-4-0-mm-3D_glrlm_HighGrayLevelRunEmphasis
	log-sigma-5-0-mm-3D_firstorder_Maximum
	log-sigma-5-0-mm-3D_firstorder_Range
	log-sigma-5-0-mm-3D_gldm_DependenceNonUniformity
	log-sigma-5-0-mm-3D_gldm_DependenceVariance
	log-sigma-5-0-mm-3D_gldm_GrayLevelNonUniformity
**Square features**	
	square_gldm_LargeDependenceHighGrayLevelEmphasis
	square_glrlm_GrayLevelNonUniformity
**Squareroot features**	
squareroot_firstorder_Mean	squareroot_firstorder_Entropy
squareroot_firstorder_Median	squareroot_firstorder_Range
squareroot_firstorder_RootMeanSquared	squareroot_glcm_Imc2
	squareroot_glcm_JointAverage
	squareroot_glcm_JointEntropy
	squareroot_glcm_SumAverage
	squareroot_glcm_SumEntropy
	squareroot_gldm_HighGrayLevelEmphasis
	squareroot_gldm_LargeDependenceLowGrayLevelEmphasis
	squareroot_glrlm_GrayLevelNonUniformity
	squareroot_glrlm_LongRunLowGrayLevelEmphasis
	squareroot_glszm_GrayLevelVariance
	squareroot_glszm_HighGrayLevelZoneEmphasis
**Wavelet features**	
wavelet-HLL_firstorder_10Percentile	wavelet-HHH_gldm_DependenceEntropy
wavelet-HLL_firstorder_90Percentile	wavelet-HHH_glrlm_ShortRunHighGrayLevelEmphasis
wavelet-HLL_firstorder_InterquartileRange	wavelet-HHH_glszm_GrayLevelNonUniformity
wavelet-HLL_firstorder_Maximum	wavelet-HHL_firstorder_10Percentile
wavelet-HLL_firstorder_MeanAbsoluteDeviation	wavelet-HHL_firstorder_90Percentile
wavelet-HLL_firstorder_Minimum	wavelet-HHL_firstorder_InterquartileRange
wavelet-HLL_firstorder_Range	wavelet-HHL_firstorder_MeanAbsoluteDeviation
wavelet-HLL_firstorder_RobustMeanAbsoluteDeviation	wavelet-HHL_firstorder_RobustMeanAbsoluteDeviation
wavelet-HLL_firstorder_Variance	wavelet-HHL_glrlm_ShortRunHighGrayLevelEmphasis
wavelet-HLL_glrlm_LowGrayLevelRunEmphasis	wavelet-HHL_glszm_SmallAreaEmphasis
wavelet-LLL_firstorder_Kurtosis	wavelet-HLH_gldm_DependenceNonUniformity
wavelet-LLL_firstorder_Mean	wavelet-HLH_glszm_SizeZoneNonUniformity
wavelet-LLL_firstorder_RootMeanSquared	wavelet-HLH_glszm_SmallAreaLowGrayLevelEmphasis
wavelet-LLL_firstorder_Skewness	wavelet-HLL_glcm_Autocorrelation
wavelet-LLL_glcm_Contrast	wavelet-HLL_glcm_JointAverage
wavelet-LLL_glcm_DifferenceAverage	wavelet-HLL_glcm_SumAverage
wavelet-LLL_glcm_DifferenceEntropy	wavelet-HLL_gldm_LowGrayLevelEmphasis
wavelet-LLL_glcm_DifferenceVariance	wavelet-HLL_glszm_GrayLevelNonUniformity
wavelet-LLL_glcm_Id	wavelet-HLL_glszm_SizeZoneNonUniformity

wavelet-LLL_glcm_Idm	wavelet-HLL_glszm_ZoneEntropy
wavelet-LLL_glcm_InverseVariance	wavelet-LHH_glszm_SizeZoneNonUniformity
wavelet-LLL_gldm_DependenceNonUniformityNormalized	wavelet-LHL_firstorder_Maximum
wavelet-LLL_gldm_LargeDependenceEmphasis	wavelet-LHL_firstorder_Minimum
wavelet-LLL_glrlm_RunLengthNonUniformity	wavelet-LHL_firstorder_Range
	wavelet-LHL_firstorder_Skewness
	wavelet-LHL_glcm_ClusterShade
	wavelet-LHL_glrlm_LongRunHighGrayLevelEmphasis
	wavelet-LHL_glszm_GrayLevelNonUniformity
	wavelet-LHL_glszm_HighGrayLevelZoneEmphasis
	wavelet-LHL_glszm_LowGrayLevelZoneEmphasis
	wavelet-LHL_glszm_SizeZoneNonUniformity
	wavelet-LHL_glszm_ZoneEntropy
	wavelet-LLH_glcm_Autocorrelation
	wavelet-LLH_glcm_JointAverage
	wavelet-LLH_glcm_JointEnergy
	wavelet-LLH_glcm_MaximumProbability
	wavelet-LLH_glcm_SumAverage
	wavelet-LLH_glcm_SumEntropy
	wavelet-LLH_gldm_DependenceVariance
	wavelet-LLH_gldm_HighGrayLevelEmphasis
	wavelet-LLH_glrlm_HighGrayLevelRunEmphasis
	wavelet-LLH_glrlm_RunLengthNonUniformity
	wavelet-LLL_glcm_Imc2
	wavelet-LLL_glrlm_LowGrayLevelRunEmphasis
	wavelet-LLL_glrlm_ShortRunLowGrayLevelEmphasis
	wavelet-LLL_glszm_HighGrayLevelZoneEmphasis
	wavelet-LLL_glszm_ZoneEntropy

Abbreviations: glcm, gray-level co-occurrence matrix; glrlm, gray-level run-length matrix; glszm, gray-level size zone matrix; gldm, gray-level dependence matrix.

Supplemental Table 5. Definition of the metrics.

**Metrics**	**Definition**
Accuracy	(TP+TN)/(TP+TN+FP+FN)
Precision	TP/(TP+FP)
Specificity	TN/(TN+FP)
Sensitivity	TP/(TP+FN)
AUC	Areas under the receiver operating characteristic curve

Abbreviations: TP, true positive; TN, true negative; FP, false positive; FN, false negative.
